# Author Correction: Engineered droplet-forming peptide as photocontrollable phase modulator for fused in sarcoma protein

**DOI:** 10.1038/s41467-024-51665-3

**Published:** 2024-08-22

**Authors:** Hao-Yu Chuang, Ruei-Yu He, Yung-An Huang, Wan-Ting Hsu, Ya-Jen Cheng, Zheng-Rong Guo, Niaz Wali, Ing-Shouh Hwang, Jiun-Jie Shie, Joseph Jen-Tse Huang

**Affiliations:** 1 Institute of Chemistry, Academia Sinica, Taipei, 115 Taiwan; 2 Chemical Biology and Molecular Biophysics, Taiwan International Graduate Program, Academia Sinica, Taipei, 115 Taiwan; 3 Department of Chemistry, National Tsing Hua University, Hsinchu, 300 Taiwan; 4 Neuroscience Program of Academia Sinica, Academia Sinica, Taipei, 115 Taiwan; 5 Institute of Molecular Biology, Academia Sinica, Taipei, 115 Taiwan; 6 Institute of Physics, Academia Sinica, Taipei, 115 Taiwan; 7 Sustainable Chemical Science and Technology, Taiwan International Graduate Program, Academia Sinica, Taipei, 115 Taiwan; 8 Department of Applied Chemistry, National Chiayi University, Chiayi City, 600 Taiwan

**Keywords:** Chemical biology, Supramolecular chemistry, Protein aggregation, Biomaterials, Biophysical chemistry

Correction to: *Nature Communications* 10.1038/s41467-024-50025-5, published online 6 July 2024

The original version of this Article contained an error in Fig. 2b, in which the image of the Eppendorf at 24 h was incorrect. The correct version of Fig. 2 is:


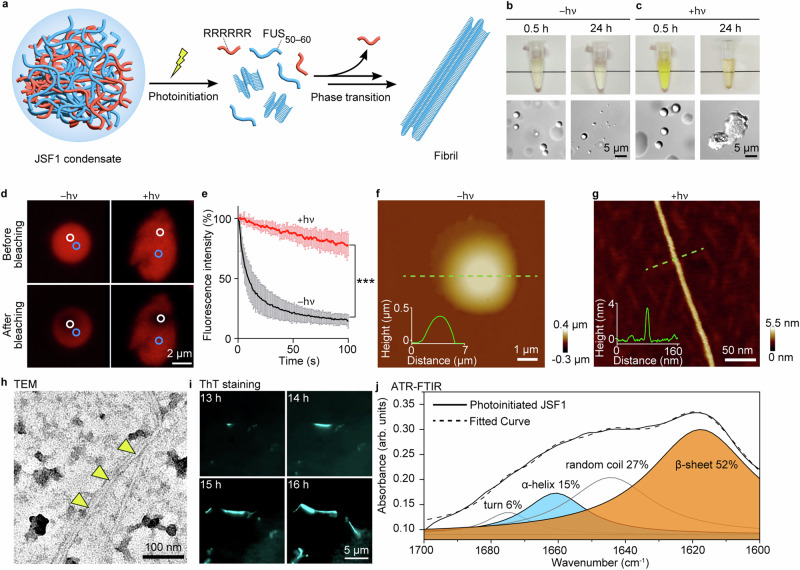


which replaces the previous incorrect version:
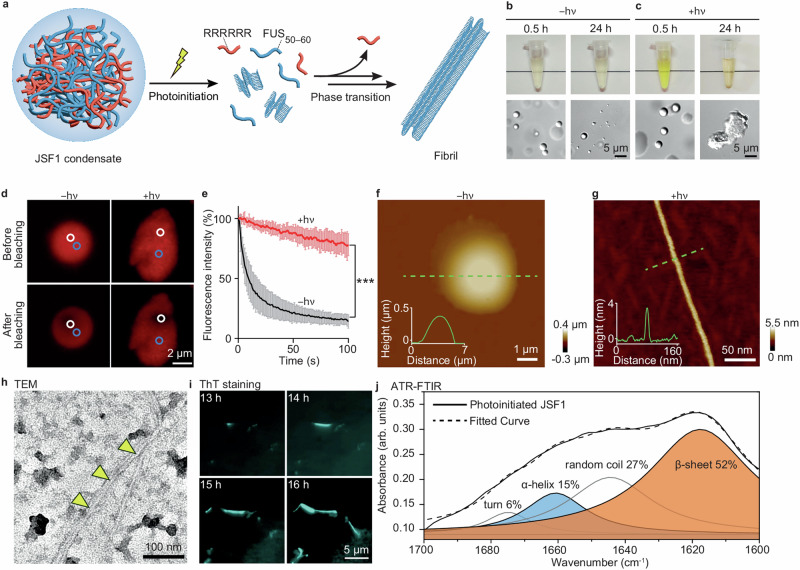


This has been corrected in both the PDF and HTML versions of the Article.

