# Supplementary material for: Engineered droplet-forming peptide as photocontrollable phase modulator for fused in sarcoma protein
Source: Nat Commun. 2024 Jul 6;15:5686. doi: 10.1038/s41467-024-50025-5 (PMC11227587; doi:10.1038/s41467-024-50025-5)
Supplement: Supplementary file 1 — Supplementary Information [file 41467_2024_50025_MOESM1_ESM.pdf]

Supplementary Information

Engineered droplet-forming peptide as photocontrollable phase modulator for fused in sarcoma protein

Hao-Yu Chuang[1,2,3], Ruei-Yu He[1], Yung-An Huang[1], Wan-Ting Hsu[1], Ya-Jen Cheng[4,5], Zheng-Rong Guo[6], Niaz Wali[1], Ing-Shouh Hwang[6], Jiun-Jie Shie[1], and Joseph Jen-Tse Huang[1,4,7,8*]

[1]Institute of Chemistry, Academia Sinica, Taipei 115, Taiwan.

[2]Chemical Biology and Molecular Biophysics, Taiwan International Graduate Program, Academia Sinica, Taipei 115, Taiwan.

[3]Department of Chemistry, National Tsing Hua University, Hsinchu 300, Taiwan.

[4]Neuroscience Program of Academia Sinica, Academia Sinica, Taipei 115, Taiwan.

[5]Institute of Molecular Biology, Academia Sinica, Taipei 115, Taiwan.

[6]Institute of Physics, Academia Sinica, Taipei 115, Taiwan.

[7]Sustainable Chemical Science and Technology, Taiwan International Graduate Program, Academia Sinica, Taipei 115, Taiwan.

[8]Department of Applied Chemistry, National Chiayi University, Chiayi City 600, Taiwan.

*e-mail: jthuang@gate.sinica.edu.tw

Table of contents **Page number**

Supplementary Method……………..……………………..…………………3

Supplementary Figures

Supplementary Fig. 1……………………………………...………………..10
Supplementary Fig. 2……………………………………...………………..10
Supplementary Fig. 3……………………………………...………………..11
Supplementary Fig. 4……………………………………...………………..11
Supplementary Fig. 5……………………………………...………………..12
Supplementary Fig. 6……………………………………...………………..12
Supplementary Fig. 7……………………………………...………………..12
Supplementary Fig. 8……………………………………...………………..13
Supplementary Fig. 9……………………………………...………………..13
Supplementary Fig. 10..…………………………………...………………..13
Supplementary Fig. 11..…………………………………...………………..14
Supplementary Fig. 12..…………………………………...………………..15
Supplementary Fig. 13..…………………………………...………………..15
Supplementary Fig. 14..…………………………………...………………..16
Supplementary Fig. 15..…………………………………...………………..16
Supplementary Fig. 16..…………………………………...………………..16
Supplementary Fig. 17..…………………………………...………………..17
Supplementary Fig. 18..…………………………………...………………..17
Supplementary Fig. 19..…………………………………...………………..17
Supplementary Fig. 20..…………………………………...………………..18
Supplementary Fig. 21..…………………………………...………………..18
Supplementary Fig. 22..…………………………………...………………..18
Supplementary Fig. 23..…………………………………...………………..19
Supplementary Fig. 24..…………………………………...………………..19
Supplementary Fig. 25..…………………………………...………………..20
Supplementary Fig. 26..…………………………………...………………..20
Supplementary Fig. 27..…………………………………...………………..20
Supplementary Fig. 28..…………………………………...………………..21

Supplementary Method

Synthetic Procedures and Product Characterization of JJS-0341.

Our approach for the synthesis of BODIPY-based fluorescent dye **JJS-0341** is shown in Scheme 1. First, 2,2'-bipyrrole **1** was obtained in 35% yield by a homocoupling reaction of pyrrole in the presence of TMSBr and PIFA. Formyl pyrrole **5** could be prepared easily from pyrrole in a 4-step sequence with an overall yield of 58%. The $POCl_3$-promoted condensation of **1** and **5**, followed by construction of the BODIPY core using $BF_3{\cdot}OEt_2$ and $i$$Pr_2NEt$ under mild reaction conditions, provided the BODIPY ethyl ester **6**. The subsequent hydrolysis was carried out under acidic conditions to provide the corresponding carboxylic acid **7**. *N*-Boc ethyl maleimide **8** was obtained in 34% yield from *N*-Boc-ethanolamine and maleimide under typical Mitsunobu reaction conditions using Ph_3P and DIAD. After removing the Boc protecting group of **8** and NHS/EDCI-activation of **7**, the *N*-hydroxysuccinimide ester product underwent a coupling reaction with amine-TFA salt **9** to give the target BODIPY-based dye **JJS-0341**.

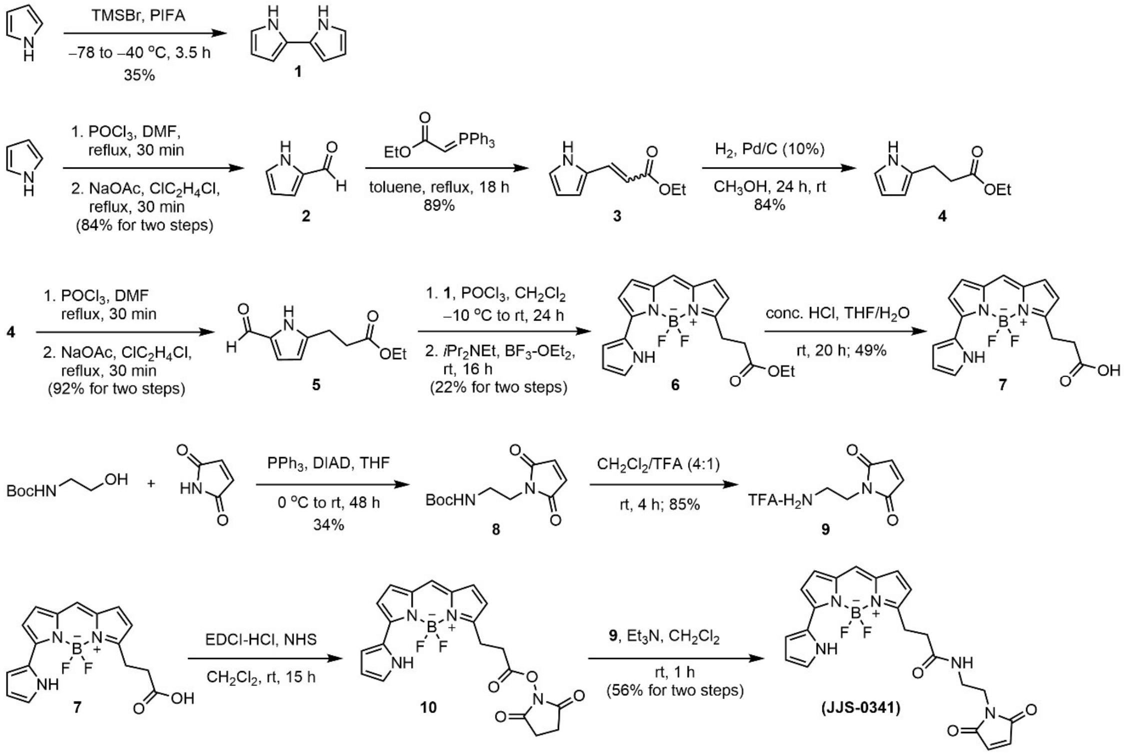

Scheme 1. Synthesis of BODIPY-based fluorescent dye (JJS-0341).

Synthesis of 1*H*,1'*H*-2,2'-Bipyrrole (1): To a solution of pyrrole (1.52 mL, 22.0 mmol) in anhydrous CH_2Cl_2 (95 mL) was added bromotrimethylsilane (TMSBr, 9 mL, 22.0 mmol) and

bis(trifluoroacetoxy)iodobenzene (PIFA, 4.73 g, 11.0 mmol) at −78 °C. After stirring for 3.5 h at −40 °C, the reaction mixture was added sat. $NaHCO_3$ solution (60 mL) at −40 °C and further stirred for 20 min at room temperature. The reaction mixture was washed with water, and the aqueous layer was extracted with CH_2Cl_2 (× 3). The organic extracts were dried over $MgSO_4$, filtered and concentrated. The residue was purified by flash column chromatography on silica gel (30% ethyl acetate in hexane) to afford the desired product **1** (1.01 g, 35%) as a white solid. $C_8H_8N_2$; mp 187–189 °C; TLC (30% ethyl acetate in hexane) R_f = 0.35; ^{1}H NMR (500 MHz, $CDCl_3$) δ 8.27 (br s, 2H), 6.75 (dd, J = 2.3, 3.6 Hz, 2H), 6.22 (dd, J = 4.0, 6.0 Hz, 2H), 6.20–6.19 (m, 2H); ^{13}C NMR (125 MHz, $CDCl_3$) δ 125.9, 117.5, 109.4, 103.6; EI-HRMS calcd for $C_8H_8N_2$: 132.0687, found: *m/z* 132.0687 $[M]^+$.

Synthesis of 1*H*-Pyrrole-2-carbaldehyde (2): Anhydrous DMF (13.8 mL, 180 mmol) was placed under a nitrogen atmosphere and cooled to 0 °C. $POCl_3$ (16.8 mL, 180 mmol) was added dropwise. The reaction mixture was allowed to room temperature for 30 min, and then dichloroethane (20 mL) was added. A solution of pyrrole (10.35 mL, 150 mmol) in dichloroethane (136 mL) was added dropwise at 0 °C. The resulting mixture was heated at reflux for 30 min before being cooled to room temperature. A solution of NaOAc (110 g, 1.35 mol) in water (140 mL) was added, and the reaction was refluxed once more for 30 min with vigorous stirring. The reaction was cooled to room temperature and extracted with CH_2Cl_2 (× 3). The combined organic extracts were dried over $MgSO_4$, filtered and concentrated. The residue was purified by flash column chromatography on silica gel (20% ethyl acetate in hexane) to afford the desired product **2** (11.98 g, 84%) as a white solid. C_5H_5NO; mp 42–44 °C; TLC (20% ethyl acetate in hexane) R_f = 0.32; ^{1}H NMR (500 MHz, $CDCl_3$) δ 10.5 (br s, 1H), 9.49 (s, 1H), 7.16–7.15 (m, 1H), 7.00–6.98 (m, 1H), 6.33 (dt, J = 3.8, 2.3 Hz, 1H) ^{13}C NMR (125 MHz, $CDCl_3$) δ 179.4, 132.8, 127.0, 121.9, 111.3; EI-HRMS calcd for C_5H_5NO: 95.0371, found: *m/z* 95.0373 $[M]^+$.

Synthesis of Ethyl 3-(1*H*-pyrrol-2-yl)acrylate (3): To a solution of **2** (1.5 g, 15.7 mmol) in

anhydrous toluene (45 mL) was added (carbethoxymethylene)triphenylphosphorane (5.49 g, 15.7 mmol). After reflux for 18 h, the reaction mixture was concentrated, and the residue was purified by flash column chromatography on silica gel (20% ethyl acetate in hexane) to afford the desired product **3** (2.3 g, 89%) as a light yellowish solid. $C_9H_{11}NO_2$; mp 54–56 °C; TLC (20% ethyl acetate in hexane) $R_f = 0.36$; ^{1}H NMR (500 MHz, CD_3OD) δ 7.49 (d, J = 15.8 Hz, 1H), 6.92 (s, 1H), 6.49 (d, J = 2.5 Hz, 1H), 6.18 (t, J = 2.9 Hz, 1H), 6.07 (d, 15.8 Hz, 1H), 4.19 (q, J = 7.0 Hz, 2H), 1.29 (t, J = 7.0 Hz, 3H); ^{13}C NMR (100 MHz, CD_3OD) δ 169.9, 136.2, 129.5, 124.1, 115.8, 111.1, 110.8, 61.1, 14.6; EI-HRMS calcd for $C_9H_{11}NO_2$: 165.0790, found: *m/z* 165.0792 $[M]^+$.

Synthesis of Ethyl 3-(1*H*-pyrrol-2-yl)propanoate (4): To a solution of **3** (2.3 g, 14.03 mmol) in anhydrous CH_3OH (50 mL) was added Pd/C (230 mg, 10% *w*/*w*). After stirring vigorously for 24 h under a hydrogen atmosphere at room temperature, the reaction mixture was filtered through Celite and the filtrate was contracted. The residue was purified by flash column chromatography on silica gel (20% ethyl acetate in hexane) to afford the desired product **4** (1.95 g, 84%) as a colorless oil. $C_9H_{13}NO_2$; TLC (20% ethyl acetate in hexane) $R_f = 0.39$; ^{1}H NMR (400 MHz, $CDCl_3$) δ 8.56 (br s, 1H), 6.65 (s, 1H), 6.09 (d, J = 2.6 Hz, 1H), 5.91 (s, 1H), 4.14 (q, J = 7.0 Hz, 2H), 2.90 (t, J = 6.6 Hz, 2H), 2.62 (t, J = 7.0 Hz, 2H), 1.25 (t, J = 7.0 Hz, 3H); ^{13}C NMR (100 MHz, $CDCl_3$) δ 173.5, 130.4, 116.4, 107.6, 104.9, 60.2, 34.1, 22.3, 13.8; EI-HRMS calcd for $C_9H_{13}NO_2$: 167.0946, found: *m/z* 167.0945 $[M]^+$.

Synthesis of Ethyl 3-(5-formyl-1*H*-pyrrol-2-yl)propanoate (5): To a cold (0 °C) stirring solution of anhydrous DMF (1.08 mL, 13.99 mmol) was added dropwise $POCl_3$ (1.3 mL, 13.99 mmol) over 30 min. After stirring for 30 min at room temperature, a solution of **4** (1.95 g, 11.66 mmol) in dichloroethane (20 mL) was added dropwise at 0 °C. The resulting mixture was heated at reflux for 30 min before being cooled to room temperature. A solution of NaOAc (6.5 g) in water (20 mL) was added and the reaction was brought once more to reflux for 30 min with vigorous stirring. The reaction was cooled to room temperature and extracted with CH_2Cl_2

(× 3). The combined organic extracts were dried over $MgSO_4$, filtered and concentrated. The combined organic extracts were dried over $MgSO_4$, filtered and concentrated. The residue was purified by flash column chromatography on silica gel (20% ethyl acetate in hexane) to afford the desired product **5** (2.09 g, 92%) as a gray solid. $C_{10}H_{13}NO_3$; mp 57–58 °C; TLC (20% ethyl acetate in hexane) $R_f = 0.25$; ^{1}H NMR (500 MHz, $CDCl_3$) δ 10.01 (br s, 1H), 9.34 (s, 1H), 6.86 (t, $J = 2.8$ Hz, 1H), 6.07 (t, $J = 2.8$ Hz, 1H), 4.14 (q, $J = 7.0$ Hz, 2H), 2.96 (t, $J = 7.0$ Hz, 2H), 2.65 (t, $J = 7.0$ Hz, 2H), 1.23 (t, $J = 7.0$ Hz, 3H); ^{13}C NMR (125 MHz, $CDCl_3$) δ 178.2, 172.5, 141.5, 132.1, 122.7, 109.4, 60.5, 33.4, 22.8, 13.9; EI-HRMS calcd for $C_{10}H_{13}NO_3$: 195.0895, found: *m/z* 195.0897 $[M]^+$.

Synthesis of 3-(2-Ethoxycarbonylethyl)-5-(2-pyrrolyl)-4,4-difluoro-4-bora-3a,4a-diaza-*s*-indacene (6): To a solution of **1** (1.0 g, 7.5 mmol) and **5** (1.46 g, 7.5 mmol) in anhydrous CH_2Cl_2 (130 mL) was added $POCl_3$ (0.7 mL, 7.5 mmol) at –10 °C. After stirring for 24 h at room temperature, the reaction mixture was treated with *N*,*N*-diisopropylethylamine (5.8 mL, 33.75 mmol) and $BF_3{\cdot}OEt_2$ (3.7 mL, 30 mmol) at 0 °C. The resulting mixture was stirred for 16 h at room temperature and then washed with brine (× 2). The aqueous layer was extracted with CH_2Cl_2 (× 3) and the combined organic extracts were dried over $MgSO_4$, filtered and concentrated. The residue was purified by flash column chromatography on silica gel (50% CH_2Cl_2 in hexane) to afford the desired product **6** (598 mg, 22%) as a purple solid. $C_{18}H_{18}BF_2N_3O_2$; mp 114–116 °C; TLC (50% CH_2Cl_2 in hexane) $R_f = 0.33$; ^{1}H NMR (500 MHz, $CDCl_3$) δ 10.4 (br s, 1H), 7.15 (dd, $J = 3.7$, 2.6 Hz, 1H), 7.01 (d, $J = 4.5$ Hz, 1H), 6.96 (s, 2H), 6.84 (d, $J = 4.5$ Hz, 1H), 6.81 (s, $J = 4.0$ Hz, 1H), 6.35 (dt, $J = 3.6$, 2.5 Hz, 1H), 6.26 (d, $J = 4.0$ Hz, 1H), 4.15 (q, $J = 7.0$ Hz, 2H), 3.32 (t, $J = 7.5$ Hz, 2H), 2.75 (t, $J = 8.0$ Hz, 2H), 1.25 (t, $J = 7.0$ Hz, 3H); ^{13}C NMR (125 MHz, $CDCl_3$) δ 172.3, 154.6, 150.3, 137.2, 133.3, 131.4, 126.0, 125.4, 123.5, 123.1, 120.0, 117.5, 115.9, 111.2, 60.3, 33.2, 23.8, 14.0; ESI-HRMS calcd for $C_{18}H_{18}BF_2N_3NaO_2$: 380.1352, found: *m/z* 380.1349 $[M + Na]^+$.

Synthesis of 3-(2-Carboxylethyl)-5-(2-pyrrolyl)-4,4-difluoro-4-bora-3a,4a-diaza-*s*-

indacene (7): To a solution of **6** (590 mg, 1.65 mmol) in THF (90 mL) and H_2O (23 mL) was slowly added conc. HCl (40 mL) at 0 °C. After stirring for 20 h at room temperature, the organic phase was separated and the aqueous phase was washed with CH_2Cl_2 (× 2). The combined organic extracts were dried over $MgSO_4$, filtered and concentrated. The residue was purified by flash column chromatography on silica gel (5% CH_3OH in CH_2Cl_2) to afford the desired product **7** (270 mg, 49%) as a deep-purple solid. $C_{16}H_{14}BF_2N_3O_2$; mp 150–152 °C; TLC (5% CH_3OH in CH_2Cl_2) $R_f = 0.15$; ^{1}H NMR (400 MHz, $CDCl_3$) δ 10.39 (br s, 1H), 7.16 (s, 1H), 7.02 (d, $J = 4.5$ Hz, 1H), 6.97 (s, 2H), 6.85 (d, $J = 4.5$ Hz, 1H), 6.81 (d, $J = 4.0$ Hz, 1H), 6.36 (dt, $J = 3.6, 2.4$ Hz, 1H), 6.28 (d, $J = 4.0$ Hz, 1H), 3.33 (t, $J = 7.5$ Hz, 2H), 2.83 (t, $J = 8.0$ Hz, 2H); ^{13}C NMR (100 MHz, $CDCl_3$) δ 178.4, 154.1, 150.7, 137.5, 133.4, 131.6, 126.0, 125.9, 123.6, 123.3, 120.4, 117.9, 116.0, 111.4, 33.1, 23.6; EI-HRMS calcd for $C_{16}H_{14}BF_2N_3O_2$: 329.1147, found: *m/z* 329.1146 $[M]^+$.

Synthesis of 1-[2-(*N*-*tert*-Butoxycarbonylamino)ethyl]-1*H*-pyrrol-2,5-dione (8): Triphenylphosphine (PPh_3, 9.5 g, 36.2 mmol) and diisopropyl azodicarboxylate (DIAD, 7.6 mL, 36.2 mmol) were dissolved in anhydrous THF (200 mL) at 0 °C and the reaction mixture was stirred for 20 min. A cloudy yellow mixture was formatted and then a solution of *N*-Boc-ethanolamine (5.83 g, 36.2 mmol) in anhydrous THF (40 mL) was added at 0 °C. After stirring for 20 min at 0 °C, maleimide (4.22 g, 43.44 mmol) was added at the same temperature. The solution changed color quickly to orange, and the clear solution was stirred for 48 h at room temperature. The solvent was removed in vacuuo, and the orange oil was incubated with hexane/diethyl ether (1:1) to remove triphenylphosphine oxide residues. The crude mixture was purified by flash column chromatography on silica gel (10% ethyl acetate in hexane) to afford the desired product **8** (2.98 g, 34%) as a white solid. $C_{11}H_{16}N_2O_4$; mp 111–112 °C; TLC (50% Et_2O in hexane) $R_f = 0.24$; ^{1}H NMR (400 MHz, $CDCl_3$) δ 6.68 (s, 2H), 4.72 (br s, 1H), 3.63 (t, $J = 5.5$ Hz, 2H), 3.30–3.29 (m, 2H), 1.37 (s, 9H); ^{13}C NMR (100 MHz, $CDCl_3$) δ 170.7, 155.9, 134.1, 79.5, 39.3, 37.9, 28.2; FAB-HRMS calcd for $C_{11}H_{17}N_2O_4$ 241.1188, found: *m/z* 241.1182

[M + H]$^+$.

Synthesis of 2-(2,5-dioxo-2,5-dihydro-1*H*-pyrrol-1-yl)ethylamine trifluoroacetic acid slat (9): To a solution of **8** (150 mg, 0.62 mmol) in CH_2Cl_2 (4 mL) was added trifluoroacetic acid (1 mL) at 0 °C. After stirring for 4 h at room temperature, the reaction mixture was concentrated and azeotrope with anhydrous CH_2Cl_2 (× 3) to afford the desired product **9** (74 mg, 85%) as a white solid. $C_6H_8N_2O_2$; mp 101–102 °C; TLC (CH_3OH/CH_2Cl_2 = 1:9) R_f = 0.26; ^{1}H NMR (400 MHz, CD_3OD) δ 6.90 (s, 2H), 3.81 (t, J = 5.6 Hz, 2H), 3.15 (t, J = 5.6 Hz, 2H); ^{13}C NMR (100 MHz, CD_3OD) δ 172.4, 135.8, 39.9, 36.3; ESI-HRMS calcd for $C_6H_9N_2O_2$ 141.0658, found: *m/z* 141.0659 [M + H]$^+$.

Synthesis of BODIPY-based fluorescent dye (JJS-0341): To a solution of **7** (50 mg, 0.15 mmol) in anhydrous CH_2Cl_2 (5 mL) was added 1-(3-dimethylaminopropyl)-3-ethylcarbodiimide hydrochloride (EDCI-HCl, 44 mg, 0.22 mmol) and *N*-hydroxy succinimide (NHS, 26 mg, 0.22 mmol). After stirring for 15 h at room temperature, the reaction mixture was diluted with CH_2Cl_2 and washed with water and brine. The combined organic extracts were dried over $MgSO_4$, filtered, concentrated and dried to afford the NHS-activated product **10**. The crude product was used in the next step without further purification.

A solution of **10** and amine-TFA **9** (42 mg, 0.30 mmol) in anhydrous CH_2Cl_2 (5 mL) was added Et_3N (0.04 mL, 0.30 mmol) at 0 °C. The reaction mixture was stirred for 1 h at room temperature and then was concentrated. The residue was purified by flash column chromatography on silica gel (20% acetone in CH_2Cl_2) to afford the desired product **JJS-0341** (38 mg, 56%) as a green-purple solid. $C_{22}H_{20}BF_2N_5O_3$; mp 114–116 °C; TLC (20% acetone in CH_2Cl_2); R_f = 0.22; ^{1}H NMR (Fig. 8a, 500 MHz, $CDCl_3$) δ 10.49 (s, 1H), 7.16 (s, 1H), 7.02 (d, J = 4.5 Hz, 1H), 6.97 (s, 1H), 6.95 (s, 1H), 6.85 (d, J = 4.5 Hz, 1H), 6.80 (d, J = 3.7 Hz, 1H), 6.64 (s, 2H), 6.36 (s, 1H), 6.24 (d, J = 3.8 Hz, 1H), 5.88 (s, 1H), 3.66 (t, J = 5.2 Hz, 2H), 3.46 (dd, J = 11.1, 5.7 Hz, 2H), 3.22 (t, J = 7.6 Hz, 2H), 2.57 (t, J = 8.0 Hz, 2H) (Supplementary Fig. 11a); ^{13}C NMR (Fig. 8b, 125 MHz, $CDCl_3$) δ 172.1, 170.9, 154.9, 150.5, 137.3, 134.1,

133.4, 131.5, 126.2, 125.9, 123.6, 123.2, 120.3, 117.9, 116.6, 111.4, 38.5, 37.4, 35.9, 24.7 (Supplementary Fig. 11b); ESI-HRMS Calc. for $C_{22}H_{20}BF_2N_5NaO_3$ 474.1519, found: *m/z* 474.1526 $[M + Na]^+$ (Supplementary Fig. 11c).

Supplementary Figures

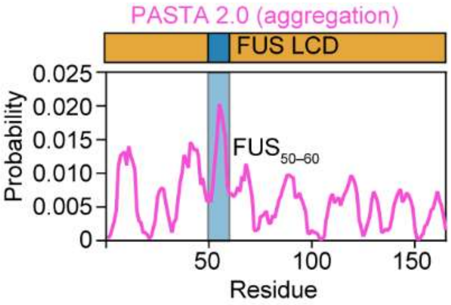

Supplementary Fig. 1. The propensity to form aggregation (using PASTA 2.0).

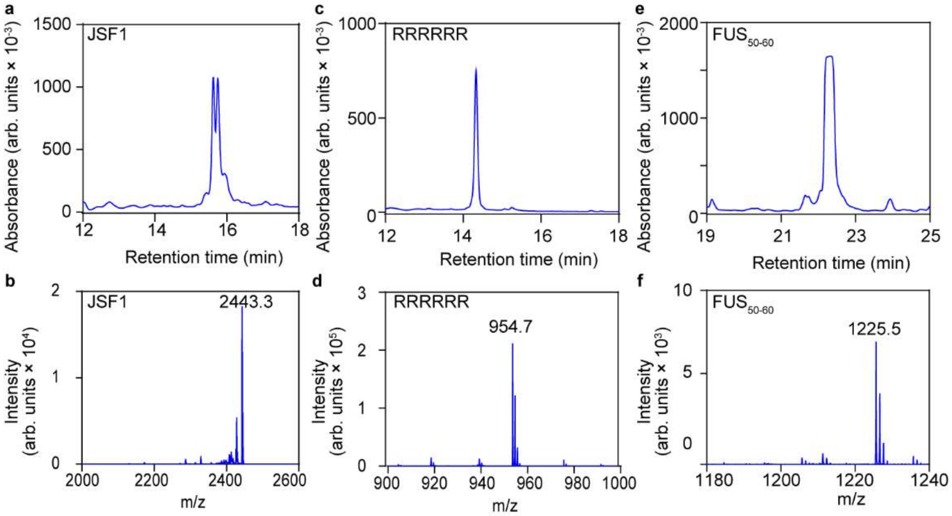

Supplementary Fig. 2. Characterization of synthesized peptides. (a) The reverse-phase high-performance liquid chromatography (RP-HPLC) results (absorbance at 220 nm) of JSF1. (b) The molecular weights of the peptides were identified by matrix-assisted laser desorption/ionization (MALDI) mass spectrum of JSF1 (Calc. 2442.62 g/mol; found m/z 2443.3 $[M + H]^+$). (c) RP-HPLC results (absorbance at 220 nm) of polyarginine tract (RRRRRR). (d) MALDI mass spectrum of polyarginine tract (Calc. 953.63 g/mol; found m/z 954.7 $[M + H]^+$). (e) RP-HPLC results (absorbance at 220 nm) of $FUS_{50\text{-}60}$. (f) MALDI mass spectrum of $FUS_{50\text{-}60}$ (Calc. 1224.50 g/mol; found m/z 1225.5 $[M + H]^+$).

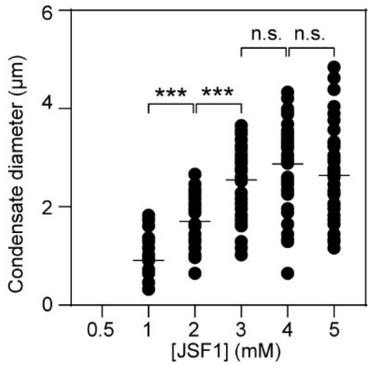

Supplementary Fig. 3. Condensates diameter of JSF1 (0.5-5 mM). The statistic results were quantified by ImageJ and shown as mean ± SD. ($n = 50$). Data were analyzed by one-way ANOVA with Tukey post-hoc test with a 95% confidence interval. ***$P < 0.001$, n.s. non-significant. 1 mM vs 2 mM: $P < 0.0001$, $q = 8.29$, $DF = 245$. 2 mM vs 3 mM: $P < 0.0001$, $q = 8.886$, $DF = 245$. 3 mM vs 4 mM: $P = 0.1205$, $q = 3.385$, $DF = 245$. 4 mM vs 5 mM: $P = 0.4086$, $q = 2.467$, $DF = 245$.

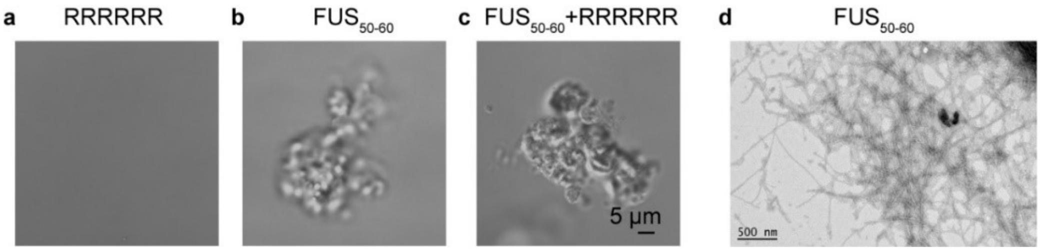

Supplementary Fig. 4. The DIC images of (a) RRRRRR (3 mM), (b) FUS_{50-60} (3 mM), and (c) FUS_{50-60} (3 mM) + RRRRRR (3 mM) and (d) TEM images of FUS_{50-60} fibrils (50 μM).

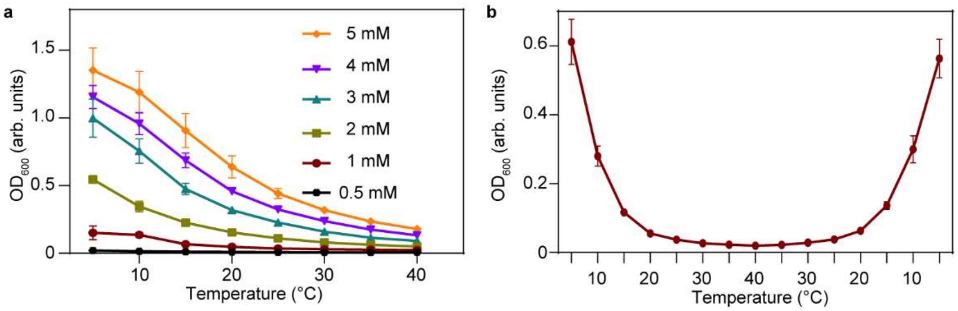

Supplementary Fig. 5. The effects of temperature on the turbidity of JSF1 solutions. (a) Turbidity of 0.5–5 mM JSF1 at 5–40 °C. (b) The temperature-dependent reversibility of 3 mM JSF1 coacervates.

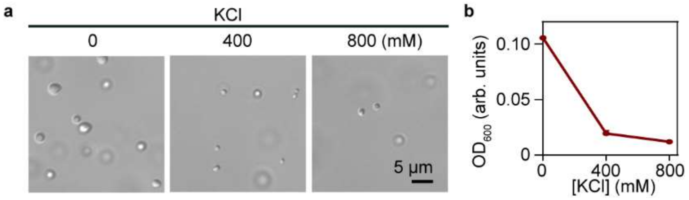

Supplementary Fig. 6. The effects of ionic strength on the LLPS of JSF1. (a) DIC images of 3 mM JSF1 with 0–800 mM of KCl. (b) Turbidity of 3 mM JSF1 with 0–800 mM of KCl.

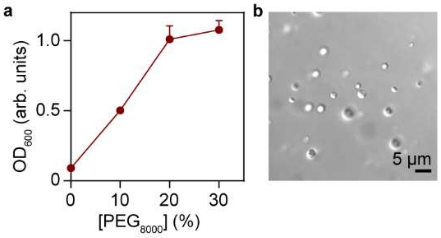

Supplementary Fig. 7. The effects of crowding agent on the LLPS of JSF1. (a) Turbidity of 3 mM JSF1 in 0–30% PEG_{8000}. (b) DIC images of 3 mM JSF1 with 30% PEG_{8000}.

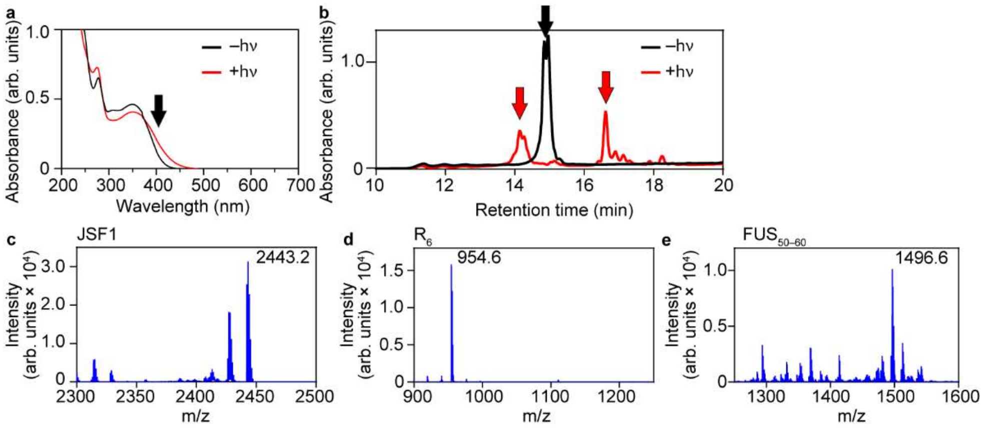

Supplementary Fig. 8. The photolysis of JSF1. (a) The UV-Vis spectra of 500 µM JSF1 with or without photoinitiation. Black arrow: the increase of absorbance at the violet light region. (b) The reverse-phase high-performance liquid chromatography results (absorbance at 220 nm) of 3 mM JSF1 before (–hν black curve) and after (+hν red curve) photoinitiation. Black arrow: peak of JSF1 –hν curve. Red arrow: peak of JSF1 +hν curve. (c)–(e) The matrix-assisted laser desorption/ionization mass spectra of peaks in Supplementary Fig. 8b. (c) JSF1 without photoinitiation at retention time =14.8 min. Calc. 2442.62 /mol; found m/z 2443.2. (d) JSF1 after photoinitiation at retention time =14.1 min (first peak). Calc. 954.15 /mol; found m/z 954.6. (e) JSF1 after photoinitiation at retention time =16.6 min (second peak). Calc. 1488.47 /mol; found m/z 1496.6.

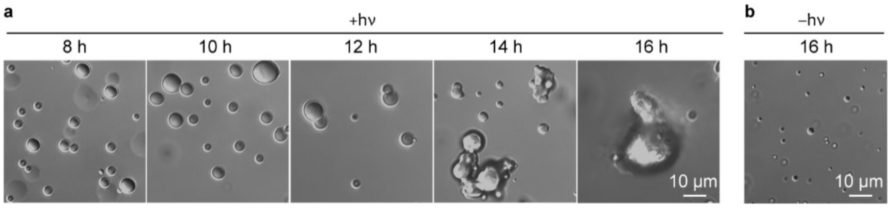

Supplementary Fig. 9. (a) DIC images of the phase transition process of photoinitiated JSF1 condensates (3 mM) after 8−16 h of incubation. (b) DIC images of JSF1 condensates (3 mM) without photoinitiation after 16 h of incubation.

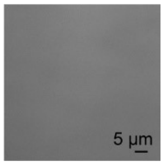

Supplementary Fig. 10. DIC images of RRRRRR (3 mM) incubated for 24 h.

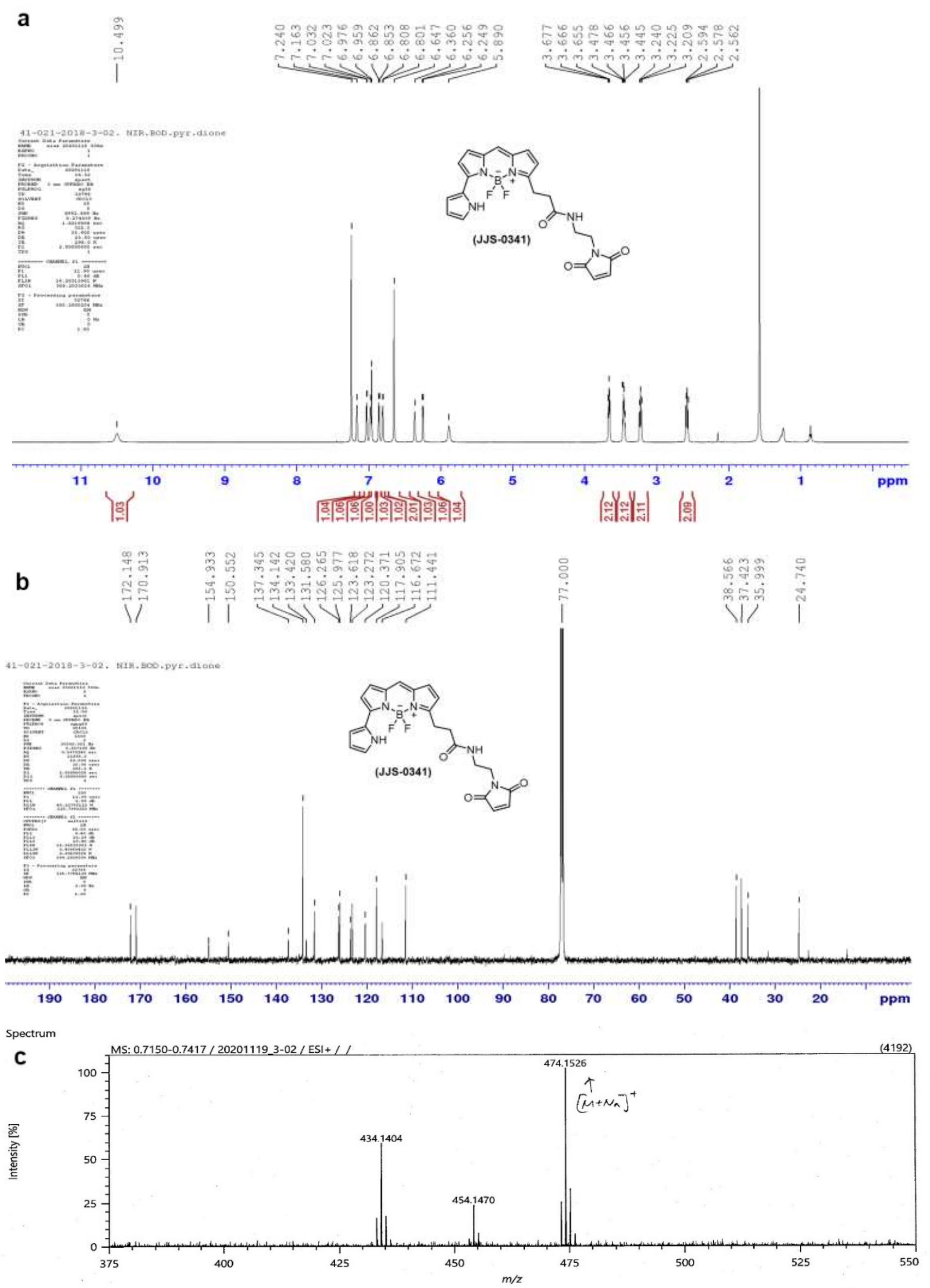

Elemental Composition

Parameters	
Tolerance:	±10.00 ppm
Electron:	Odd/Even
Charge:	+1
DBE:	-99.0 - 999.0

Elements Set 1:

Symbol	C	H	O	N	B	F	Na
Min	0	0	3	5	1	2	1
Max	1000	4000	3	5	1	2	1

Results

Mass	Formula	Calculated Mass	Mass Difference [mDa]	Mass Difference [ppm]	DBE
474.15265	C22 H20 B N5 O3 F2 Na	474.15195	0.70	1.47	14.5

Supplementary Fig. 11. The characterization of 3-((2*Z*)-2-{[1-(Difluoroboryl)-5-1*H*-pyrrol-2-yl-1*H*-pyrrol-2-yl]methylene}-2*H*-pyrrol-5-yl)-*N*-[2-(2,5-dioxo-2,5-dihydro-1*H*-pyrrol-1-yl)ethyl]propenamide (JJS-0341). (a) ^{1}H NMR (500 MHz, $CDCl_3$) δ 10.49 (s, 1H), 7.16 (s, 1H), 7.02 (d, J = 4.5 Hz, 1H), 6.97 (s, 1H), 6.95 (s, 1H), 6.85 (d, J = 4.5 Hz, 1H), 6.80 (d, J = 3.7

Hz, 1H), 6.64 (s, 2H), 6.36 (s, 1H), 6.24 (d, *J* = 3.8 Hz, 1H), 5.88 (s, 1H), 3.66 (t, *J* = 5.2 Hz, 2H), 3.46 (dd, *J* = 11.1, 5.7 Hz, 2H), 3.22 (t, *J* = 7.6 Hz, 2H), 2.57 (t, *J* = 8.0 Hz, 2H). (b) ^{13}C NMR (125 MHz, $CDCl_3$) δ 172.1, 170.9, 154.9, 150.5, 137.3, 134.1, 133.4, 131.5, 126.2, 125.9, 123.6, 123.2, 120.3, 117.9, 116.6, 111.4, 38.5, 37.4, 35.9, 24.7. (c) ESI-HRMS Calc. for $C_{22}H_{20}BF_2N_5NaO_3$ 474.1519, found: *m/z* 474.1526 $[M + Na]^+$.

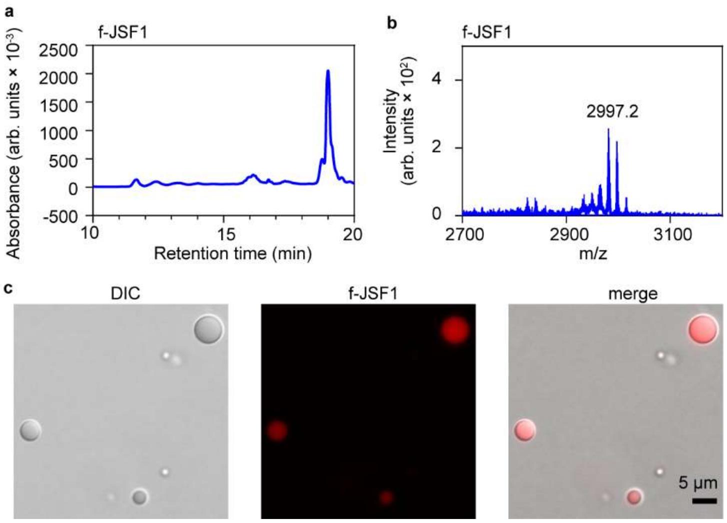

Supplementary Fig. 12. (a) RP-HPLC results (absorbance at 220 nm) of f -JSF1. (b) MALDI mass spectrum of f-JSF1 (Calc. 2995.62 g/mol; found m/z 2997.2 $[M + H]^+$). (c) 3 mM JSF1 condensates spiked with f -JSF1 (f -JSF1:JSF1 = 1:2500).

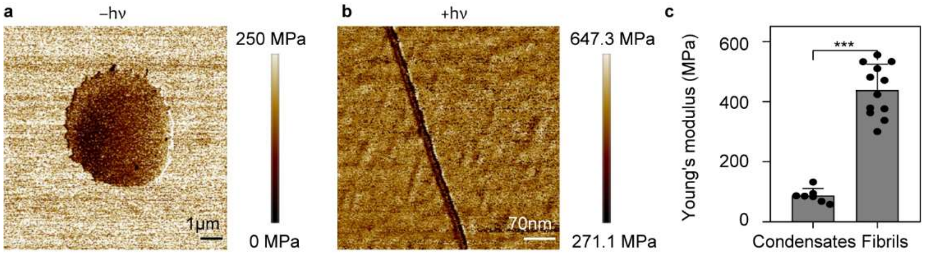

Supplementary Fig. 13. Maps of Young's modulus acquired by PeakForce Quantitative Nano-Mechanics mode along with Fig. 2f (a) and Fig. 2g (b). (c) Comparison of the Young's modulus. The statistic results were shown as mean ± SD (n ≥ 7). Data were analyzed by unpaired t test (two-tailed) using Welch's correction with a 95% confidence interval. ***P < 0.001. Condensate vs Fibrils: P < 0.0001, q = 13.27, DF = 13.55.

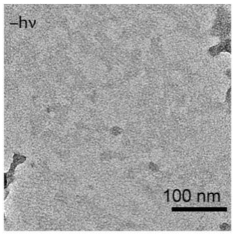

Supplementary Fig. 14. TEM image of JSF1 (3 mM) without photoinitiation incubated for 24 hours.

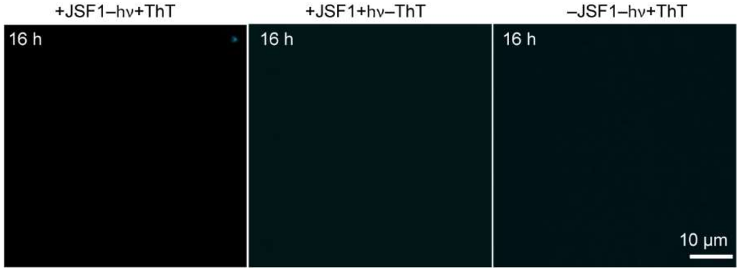

Supplementary Fig. 15. TIRF image of the controls without photoinitiation, without ThT, and ThT only for 16 h.

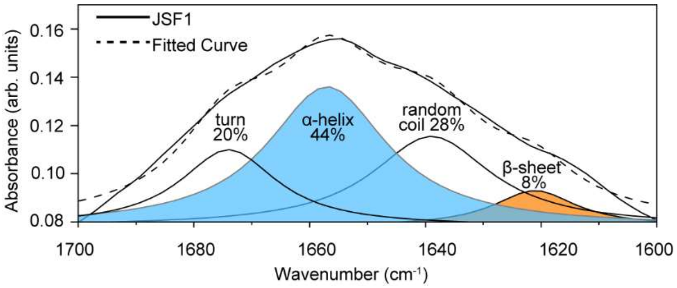

Supplementary Fig. 16. ATR-FTIR and deconvolution results of 3 mM JSF1 without photoinitiation.

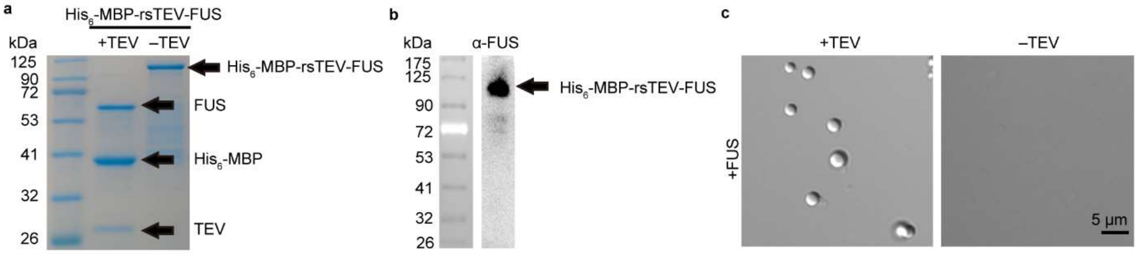

Supplementary Fig. 17. (a) FUS protein with TEV cleavage (left lane) and without TEV cleavage (right lane) checked by SDS-PAGE. rsTEV: TEV recognition site. (b) Western blot of purified Hi6-MBP-rsTEV-FUS. The anti-FUS antibody was used. (c) FUS condensates was generated after cleavage by TEV. The source data are provided as a Source Data file.

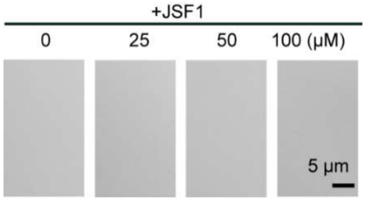

Supplementary Fig. 18. DIC images of JSF1 (0–100 μM) without FUS.

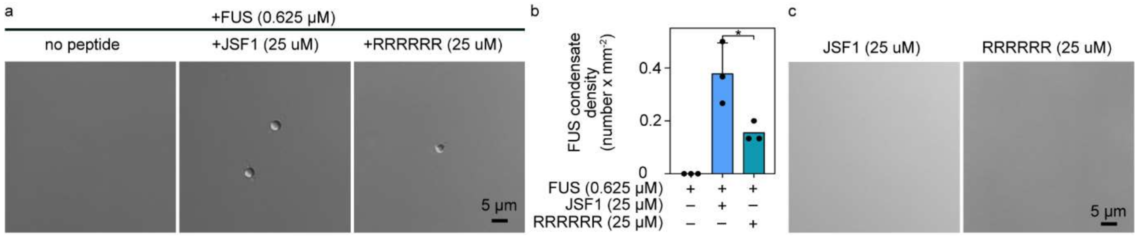

Supplementary Fig. 19. JSF1 and RRRRRR lowered the critical concentration of FUS LLPS. (a) DIC images of FUS solution (0.625 μM), with JSF1 (25 μM), and with RRRRRR (25 μM). (b) The condensate density of FUS. The statistic results were shown as mean ± SD of 3 biological replicates (n = 3). Data were analyzed by unpaired t test (two-tailed) with a 95% confidence interval. *P < 0.05. JSF1 vs RRRRRR: P = 0.0354, t = 3.123, DF = 4. (c) DIC images of JSF1 solution (25 μM) and RRRRRR solution (25 μM) without FUS.

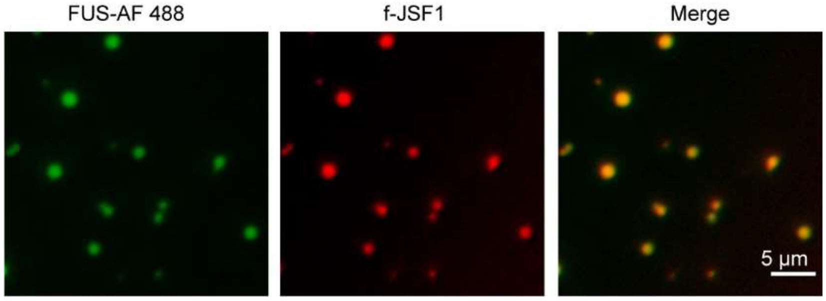

Supplementary Fig. 20. The epifluorescence images of the colocalization of FUS-AF488 and JSF1 (2.5 μM, f-JSF1:JSF1 = 1:2500).

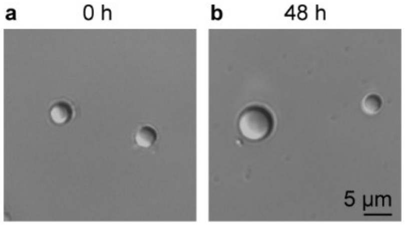

Supplementary Fig. 21. The DIC images of FUS (2.5 μM) + RRRRRR (25 μM) condensates incubated for (a) 0 h and (b) 48 h.

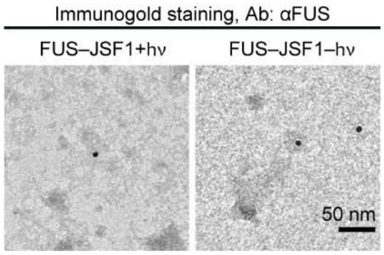

Supplementary Fig. 22. TEM images with immunogold labeling of FUS incubated with or without photoinitiation in the absence of JSF1 at 48 hours.

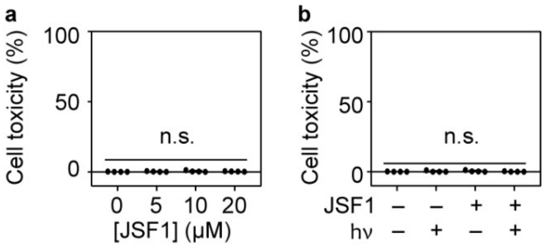

Supplementary Fig. 23. The cell toxicity of N2A cells (a) treated with different concentration (0–20 μM) of JSF1 and (b) cells in the presence or absence of JSF1 (10μM) with or without photoinitiation. The statistic results were shown as mean ± SD of 4 biological replicates (n = 4). Data were analyzed by one-way ANOVA using Tukey post-hoc test with a 95% confidence level. n.s.: non-significant. 0 μM vs 5 μM: P = 0.9060, q = 0.9490, DF = 12. 0 μM vs 10 μM: P = 0.3440, q = 2.469, DF = 12. 0 μM vs 20 μM: P = 0.7078, q = 1.527, DF = 12. –JSF1–hν vs –JSF1+hν: P = 0.8777, q = 1.051, DF = 12. –JSF1–hν vs +JSF1–hν: P = 0.5272, q = 1.968, DF = 12. +JSF1–hν vs +JSF1+hν: P = 0.6177, q = 1.747, DF = 12.

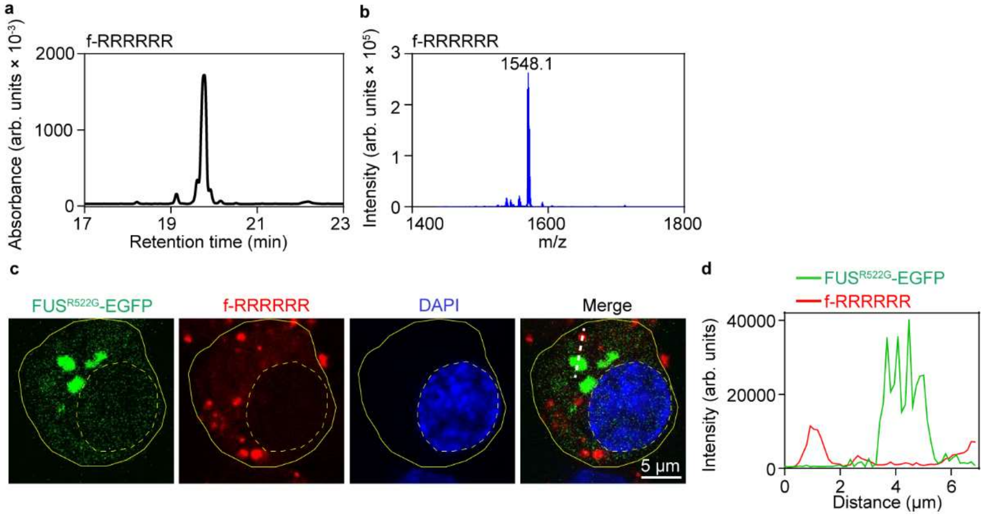

Supplementary Fig. 24. (a) The RP-HPLC results of fluorophore-attached polyarginine tract (denoted as f-RRRRRR). (b) The MALDI mass spectrum of f-RRRRRR (Calc. 1547.39 g/mol; found m/z 1548.1 $[M + H]^+$). (c) f -RRRRRR could not colocalize with FUS^{R522G}-EGFP condensates in cells. The section along the dashed line is shown in (d).

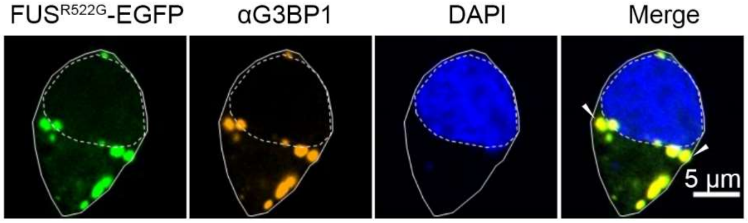

Supplementary Fig. 25. The colocalization of FUS^{R522G}-EGFP condensates with stress granule marker G3BP1 in the JSF1-treated cells. Solid line: cell. Dashed line: nucleus. White arrow: FUS^{R522G}-EGFP-containing stress granules.

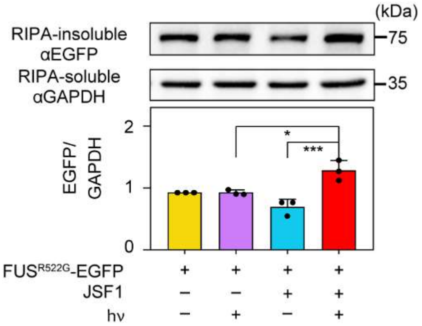

Supplementary Fig. 26. Western blot of FUS^{R522G}-EGFP harboring N2A cells in the JSF1-treated or control cells in the presence and absence of photoinitiation. The blots of RIPA-insoluble and RIPA-soluble cell lysates were demonstrated by SDS-PAGE and probed with EGFP and GAPDH antibodies. Quantification of blots of EGFP (EGFP/GAPDH) was shown below. The statistic results were quantified by ImageJ and shown as mean ± SD of 3 biological replicates (n = 3). Data were analyzed by one-way ANOVA using Tukey post-hoc test with a 95% confidence interval. *$P < 0.05$, ***$P < 0.001$. –JSF1+hν vs +JSF1+hν: $P = 0.0142$, $q = 5.824$, DF = 8. +JSF1–hν vs +JSF1+hν: $P = 0.0006$, $q = 9.710$, DF = 8. Source data are provided as a Source Data file.

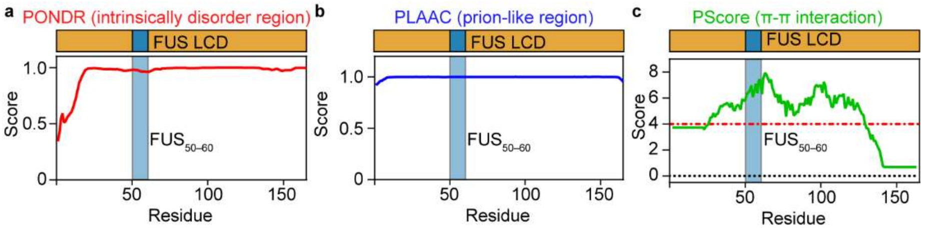

Supplementary Fig. 27. Sequence analysis of FUS LCD (1-165). (a) The prediction of intrinsically disorder (using PONDR). (b) The similarity to prion-like proteins (using PLAAC) (c) The propensity to form π-π interaction (using PScore). Red dashed line: confidence threshold. Black dashed line: PDB average.

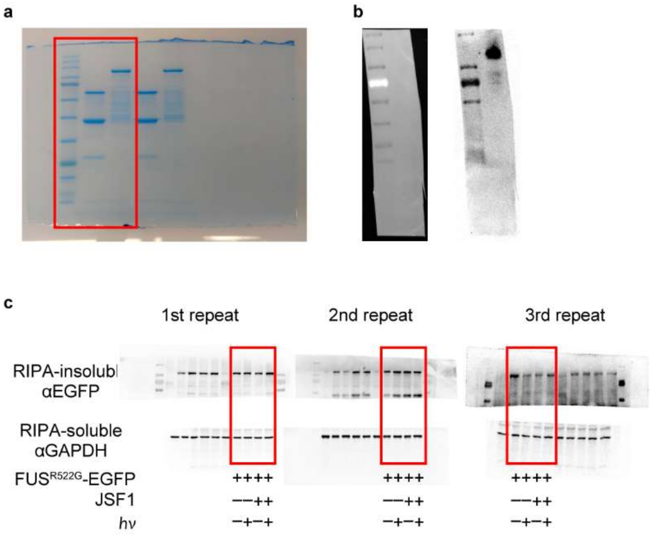

Supplementary Fig. 28. Uncropped scan of Supplementary Fig. (a) 17a, (b) 17b, and (c) Fig. 26. The groups used in this study are marked with red boxes. The internal control and the upper part were from the same membrane and separated for staining with different antibodies. The source data are provided as a Source Data file.
