# Supplementary material for: Engineered droplet-forming peptide as photocontrollable phase modulator for fused in sarcoma protein
Source: Nat Commun. 2024 Jul 6;15:5686. doi: 10.1038/s41467-024-50025-5 (PMC11227587; doi:10.1038/s41467-024-50025-5)
Supplement: Supplementary file 4 — Description of Additional Supplementary Files [file 41467_2024_50025_MOESM4_ESM.pdf]

Supplementary Movie 1:
The fusion between JSF1 condensates in 3 mM solution observed by DIC microscopy, shown in Fig. 1f.

Supplementary movie 2:
TIRF microscopy of photoinitiated JSF1 after 12-16 hours incubation in the presence of ThT, shown in Fig. 2i.
