# Supplementary material for: Engineered droplet-forming peptide as photocontrollable phase modulator for fused in sarcoma protein
Source: Nat Commun. 2024 Jul 6;15:5686. doi: 10.1038/s41467-024-50025-5 (PMC11227587; doi:10.1038/s41467-024-50025-5)
Supplement: Supplementary file 6 — Reporting Summary [file 41467_2024_50025_MOESM6_ESM.pdf]

nature portfolio

Corresponding author(s): Joseph Jen-Tse Huang

Last updated by author(s): Jun 24, 2024

Reporting Summary

Nature Portfolio wishes to improve the reproducibility of the work that we publish. This form provides structure for consistency and transparency in reporting. For further information on Nature Portfolio policies, see our Editorial Policies and the Editorial Policy Checklist.

Statistics

For all statistical analyses, confirm that the following items are present in the figure legend, table legend, main text, or Methods section.

n/a	Confirmed	
☐	☒	The exact sample size (*n*) for each experimental group/condition, given as a discrete number and unit of measurement
☐	☒	A statement on whether measurements were taken from distinct samples or whether the same sample was measured repeatedly
☐	☒	The statistical test(s) used AND whether they are one- or two-sided<br>*Only common tests should be described solely by name; describe more complex techniques in the Methods section.*
☐	☒	A description of all covariates tested
☐	☒	A description of any assumptions or corrections, such as tests of normality and adjustment for multiple comparisons
☐	☒	A full description of the statistical parameters including central tendency (e.g. means) or other basic estimates (e.g. regression coefficient) AND variation (e.g. standard deviation) or associated estimates of uncertainty (e.g. confidence intervals)
☐	☒	For null hypothesis testing, the test statistic (e.g. *F*, *t*, *r*) with confidence intervals, effect sizes, degrees of freedom and *P* value noted<br>*Give P values as exact values whenever suitable.*
☒	☐	For Bayesian analysis, information on the choice of priors and Markov chain Monte Carlo settings
☒	☐	For hierarchical and complex designs, identification of the appropriate level for tests and full reporting of outcomes
☒	☐	Estimates of effect sizes (e.g. Cohen's *d*, Pearson's *r*), indicating how they were calculated

Our web collection on statistics for biologists contains articles on many of the points above.

Software and code

Policy information about availability of computer code

Data collection	No software was used.
Data analysis	Zen, Origin, Fiji, Prism, and Nanoscope Analysis (AFM).

For manuscripts utilizing custom algorithms or software that are central to the research but not yet described in published literature, software must be made available to editors and reviewers. We strongly encourage code deposition in a community repository (e.g. GitHub). See the Nature Portfolio guidelines for submitting code & software for further information.

Data

Policy information about availability of data

All manuscripts must include a data availability statement. This statement should provide the following information, where applicable:

- Accession codes, unique identifiers, or web links for publicly available datasets
- A description of any restrictions on data availability
- For clinical datasets or third party data, please ensure that the statement adheres to our policy

The authors declare that all data supporting the findings of this study are available within the manuscript and its supplementary information files (Supplementary Information.pdf/Supplementary Movie 1 Time-lapse fusion video of JSF1 condensates.avi/Supplementary Movie 2 Time-lapse TIRF video of photoinitiated JSF1.mp4).

Research involving human participants, their data, or biological material

Policy information about studies with human participants or human data. See also policy information about sex, gender (identity/presentation), and sexual orientation and race, ethnicity and racism.

Reporting on sex and gender	Not applicable.
Reporting on race, ethnicity, or other socially relevant groupings	Not applicable.
Population characteristics	Not applicable.
Recruitment	Not applicable.
Ethics oversight	Not applicable.

Note that full information on the approval of the study protocol must also be provided in the manuscript.

Field-specific reporting

Please select the one below that is the best fit for your research. If you are not sure, read the appropriate sections before making your selection.

- [x] Life sciences
- [ ] Behavioural & social sciences
- [ ] Ecological, evolutionary & environmental sciences

For a reference copy of the document with all sections, see nature.com/documents/nr-reporting-summary-flat.pdf

Life sciences study design

All studies must disclose on these points even when the disclosure is negative.

Sample size	More than 40 condensates were counted and analyzed in each group in Fig. 4d and 4e.
Data exclusions	No data were excluded.
Replication	Three independent biological replicates were carried out for experiments with statistical analysis.
Randomization	Not relevant to our study.
Blinding	Not relevant to our study.

Reporting for specific materials, systems and methods

We require information from authors about some types of materials, experimental systems and methods used in many studies. Here, indicate whether each material, system or method listed is relevant to your study. If you are not sure if a list item applies to your research, read the appropriate section before selecting a response.

Materials & experimental systems

n/a	Involved in the study	
[ ]	[x]	Antibodies
[ ]	[x]	Eukaryotic cell lines
[x]	[ ]	Palaeontology and archaeology
[x]	[ ]	Animals and other organisms
[x]	[ ]	Clinical data
[x]	[ ]	Dual use research of concern
[x]	[ ]	Plants

Methods

n/a	Involved in the study	
[x]	[ ]	ChIP-seq
[x]	[ ]	Flow cytometry
[x]	[ ]	MRI-based neuroimaging

Antibodies

Antibodies used

anti-TLS/FUS antibody (Abcam, ab84078) (1:20 for immunogold staining, 1:1000 for WB)
anti-GFP antibody (Abcam, ab183734 (1:1000 for WB)
anti-GAPDH antibody (GeneTex, GTX627408) (1:15000 for WB)
anti-G3BP1 antibody (Abcam, ab181150) (1:5000 for immune staining)
Donkey Anti-Rabbit IgG H&L (6nm Gold) preadsorbed (Abcam, ab105294) (1:20 for immunogold staining)
Goat Anti-Rabbit IgG antibody (HRP) (GeneTex, GTX213110-01) (1:15000 for WB)

	Peroxidase AffiniPure Goat Anti-Mouse IgG (H+L)(Jackson ImmunoResearch Laboratories, Inc., 115-035-003) (1:15000 for WB)<br>Goat anti-Rabbit IgG (H+L) Cross-Adsorbed Secondary Antibody, Alexa Fluor™ 568 (A-11011) (1:5000 for immune staining)
Validation	For all validations of antibodies could be found on the following websites:<br>https://www.abcam.com/products/primary-antibodies/tlsfus-antibody-ab84078.html<br>https://www.abcam.com/products/primary-antibodies/gfp-antibody-epr14104-ab183734.html<br>https://www.genetex.com/Product/Detail/GAPDH-antibody-GT239/GTX627408<br>https://www.abcam.com/products/primary-antibodies/g3bp-antibody-epr13986b-ab181150.html<br>https://www.abcam.com/products/secondary-antibodies/donkey-rabbit-igg-hl-6nm-gold-preadsorbed-ab105294.html<br>https://www.genetex.com/Product/Detail/Goat-Anti-Rabbit-IgG-antibody-HRP/GTX213110-01<br>https://www.jacksonimmuno.com/catalog/products/115-035-003<br>https://www.thermofisher.com/antibody/product/Goat-anti-Rabbit-IgG-H-L-Cross-Adsorbed-Secondary-Antibody-Polyclonal/A-11011

Eukaryotic cell lines

Policy information about cell lines and Sex and Gender in Research

Cell line source(s)	N2A
Authentication	N2A was a gift from Dr. Yijuang Chern (Institute of Biomedical Sciences, Academia Sinica, Taiwan) through collaboration.
Mycoplasma contamination	These cell lines were not tested by PCR; however, DAPI staining confirmed little or none Mycoplasma.
Commonly misidentified lines<br>(See ICLAC register)	Not applicable.

Plants

Seed stocks	Not applicable.
Novel plant genotypes	Not applicable.
Authentication	Not applicable.
